# Association of acetabular implants with sensitive radiographic surveillance on revision rates: a study based on 5 hip arthroplasty registries

**DOI:** 10.2340/17453674.2026.45292

**Published:** 2026-02-03

**Authors:** Chan Hee CHO, John M ABRAHAMS, Deepti K SHARMA, Lucian B SOLOMON, Christopher J WALL, Bart G PIJLS, Stuart A CALLARY

**Affiliations:** 1 Centre for Orthopaedic and Trauma Research, Faculty of Health and Medical Sciences, The University of Adelaide, Adelaide, SA; 2 Department of Orthopaedics and Trauma Royal Adelaide Hospital, Adelaide, SA; 3 Australian Orthopaedic Association National Joint Replacement Registry, Adelaide, SA, Australia; 4 Department of Orthopaedics, Leiden University Medical Centre, Leiden, Netherlands

## Abstract

**Background and purpose:**

National joint arthroplasty registries are the gold standard for monitoring long-term acetabular implant survivorship. Sensitive radiographic surveillance (SRS) has been recommended as a complementary surveillance approach, but no study has investigated whether implants introduced with no sensitive radiographic surveillance (NSRS) are associated with higher revision rates. Therefore, we investigated whether acetabular implants with NSRS are associated with higher revision rates than those with SRS.

**Methods:**

Acetabular implants with SRS were defined as those with published evidence of stability measurements assessed using either radiostereometric analysis or “Ein Bild Röntgen Analyse.” Evidence of SRS of acetabular implant designs was sourced from 2 literature reviews. A mixed-effects model was used to pool and compare the revision rate of acetabular implants with SRS and NSRS at 5 and 10 years from 5 arthroplasty registries.

**Results:**

There were 29 unique acetabular implant designs with SRS and 86 designs with NSRS that had matching 5- and 10-year revision rates. At 5 years, there was a mean difference of 0.8% (95% confidence interval [CI] 0.5–1.1) in mean all-cause revision rates favoring implants with SRS. Mean all-cause revision rates at 10 years for acetabular implants with SRS and NSRS were 5.2% (CI 4.9–5.5) and 7.4% (CI 7.0–7.9) respectively, with a mean difference of 1.8% (CI 1.2–2.3) favoring implants with SRS.

**Conclusion:**

Acetabular implants with NSRS were associated with 1.8% higher pooled revision rates than those with SRS at 10 years, which represents a relative increase in acetabular revision burden of approximately 36%.

National and regional arthroplasty registries are considered the gold standard for monitoring orthopedic implant performance. While awaiting national registry long-term survivorship outcomes, smaller clinical trials and single-center cohort studies use other complementary surveillance methods including complication rates, patient-reported outcome measures, and radiographic measurements of implant stability during the phased introduction of new implants. Monitoring patient outcomes to establish safety and efficacy of surgical interventions should be considered standard practice. A systematic review found that 24% of all total hip arthroplasty (THA) implants clinically available in the United Kingdom had no published evidence of clinical effectiveness [1]. This was in part due to a historical rule that stated new implant designs do not need further clearance by regulatory entities if they were similar to a previous acceptable design. Disappointingly, minor implant design changes have led to early catastrophic failures in the past [2]. With the recent changes in the European Union Medical Device Regulation (EUMDR), clinical evidence and surveillance of implant performance are now mandatory in the European Union prior to widespread usage of new designs [3].

Measuring early implant stability relative to the surrounding bone, using either radiostereometric analysis (RSA) or “Ein Bild Röntgen Analyse” (EBRA), is the only validated surrogate marker for predicting long-term outcomes in primary THA [4]. Early migration of acetabular implants has been associated with later loosening, allowing for the early identification of underperforming implants [5]. While recommended [6-8], sensitive radiographic surveillance (SRS) remains optional for new acetabular implant designs during their introduction. Migration data from RSA and EBRA studies provide early feedback on implant performance, allowing evidence-based decision-making regarding implant introduction. Acetabular implants that have undergone SRS and have been found to have low early migration may provide clinicians with confidence in the continued use of that implant. In the absence of published evidence of SRS as an early surrogate outcome, clinicians may have to wait for evidence of longer-term clinical success of acetabular implants. Therefore, the aim of our study was to investigate whether implants with no SRS (NSRS) are associated with higher revision rates than those with SRS.

## Methods

### Study design

This observational study is reported according to the STROBE guidelines.

### Registry search

National and regional hip arthroplasty registries were identified through the Network Orthopaedic Registries of Europe – European Federation of National Associations of Orthopaedics and Traumatology (NORE-EFORT) webpage [9] and a previous study of RSA-tested total knee arthroplasty (TKA) implants [10]. Data was collected in September 2023 and the latest publicly available annual reports were extracted from each registry. The inclusion criterion of this study was any registry annual report that reported the revision data of acetabular implants following a primary THA with a minimum follow-up of 10 years for each implant. Hip arthroplasty registries were excluded if they did not report 10-year follow-up. Implants with a low number of cases were more likely to be used in rare surgical cases or were newer protheses that lacked 10-year revision rate data. Therefore, registry survivorship results of acetabular implants with less than 250 cases were excluded from further analysis.

### Identification of acetabular implants with SRS

Acetabular implants with SRS were defined as those that had published evidence of migration measured using either RSA or EBRA. Acetabular implants with NSRS were defined as those that had no published evidence of migration measured using either RSA or EBRA. Both RSA and EBRA are the only validated surrogate markers for predicting long-term outcomes in primary THA [4] and enable sensitive measurements to establish the mean implant migration of a cohort with a relatively small number of patients. Acetabular implants studied with RSA were identified by a recently published systematic review [11]. To identify acetabular implants studied with EBRA, a systematic search was conducted in PubMed, Embase, and Scopus databases (Appendix 1). Covidence systematic review management software (https://www.covidence.org/) was used to export the searches to conduct a blinded review. Title, abstract, and full text screening were performed by 2 reviewers (CC and JA, Appendix 2 and 3). 3 additional unique acetabular implant designs (Conserve Plus, Duraloc, and Trident) were added from the EBRA study to the acetabular implants identified in the RSA review [12-14].

### Data matching and extraction

Acetabular implants with SRS were identified from both reviews and matched to the 5- and 10-year registry revision data. Data that was extracted included the model of acetabular implants, fixation method of the acetabular implants, year of publication, and the model of its corresponding femoral stem. The registries did not consistently report the revision rates of individual acetabular implants but rather reported the revision rates of acetabular and femoral implant combinations. For example, the registries reported the revision rate for the Trident acetabular shell/Exeter V40 stem combination and a separate revision rate for the Trident acetabular shell/Accolade II stem combination. However, most registries did not report the revision rate for the acetabular implant alone. Therefore, analysis of the revision rate of acetabular implants in isolation was not possible. We thus performed 2 separate analyses, comparing the registry-based survivorship data for (i) matching implants according to the cup and stem combination as described in SRS studies to implant combinations in registry reports and (ii) matching cup type alone, irrespective of the cup and stem combination shown in registry reports. Our study investigated only the migration of acetabular implants because the RSA and EBRA studies identified in the reviews only reported the migration of the acetabular implants. A secondary analysis of acetabular implants with evidence of SRS was then limited to matching stem designs used in EBRA and RSA studies, where reported. This was performed to investigate the influence of different stem designs on the pooled revision rates.

All acetabular implant designs reported in the included hip arthroplasty registries were separated into 2 groups: SRS and NSRS. The all-cause revision rates with their corresponding 95% confidence intervals (CI) or standard error (SE) at 5 and 10 years were extracted from every registry report. The 95% CIs were then converted into an SE [15]. As RSA and EBRA measurements are primarily used for the early detection of implant loosening, it would have been preferable to extract data concerning revision due to loosening. However, the registries did not consistently report the revision data due to loosening in publicly available data and therefore only all-cause revision data was used as the primary outcome measure. This is consistent with previous investigations in TKA [10] and reflective of the all-cause revision rates typically available to clinicians and policymakers when evaluating implant performance outside of specific registry collaborations.

### Statistics

A mixed-effects model was used to calculate the pooled all-cause revision at 5- and 10-years’ follow-up for THA designs with SRS and NSRS with a random effect for pooling of revision rate and the variable coding for SRS or NSRS as a moderator. This was repeated to analyze acetabular implants with SRS and matched stems against acetabular implants with NSRS. The DerSimonian–Laird estimator was used to account for the heterogeneity between the THA designs with NSRS and SRS [10]. Pooled all-cause revision percentages were calculated separately for each registry for all THA designs with NSRS and SRS. The results of this study are presented in revision rate percentages (%) with their corresponding 95% CI. Metafor package (version 3.4-0) in R-Studio (R version 4.2.1; R Foundation for Statistical Computing, Vienna, Austria) was used for all data analysis in this study [16]. To avoid the bias associated with dichotomous interpretations of P values, our findings are presented with 95% CIs to convey the full magnitude of the results [17].

### Ethics, registration, data sharing, use of AI tools, funding, and disclosure

No ethical approval was required for this study as the data was retrieved from previous published studies. This study did not have any prior registrations. The study was not funded by any external or internal party. No AI-assisted tools were used in the preparation or submission of this work. There are no conflicts of interest for any of the authors. Complete disclosure of interest forms according to ICMJE are available on the article page, doi: 10.2340/17453674.2026.45292

## Results

The registry search identified 31 national or regional arthroplasty registries, of which 5 arthroplasty registries were included in the pooled analysis (Australia [18], Netherlands [19], Finland [20], United Kingdom [21], and Emilia Romagna [22]) (Figure). The United Kingdom had the greatest number of registered THA (n = 1,614,242) and Emilia Romagna had the least (n = 137,612) (Table 1). Across the 5 included registries, the number of acetabular and stem implant combinations with 5-year and 10-year revision data ranged from 40 to 93 and the number of acetabular implant designs ranged from 26 to 51 (Tables 2 and 3). There were 29 unique acetabular implant designs with SRS and 82 acetabular implant designs with NSRS.

**Figure:**
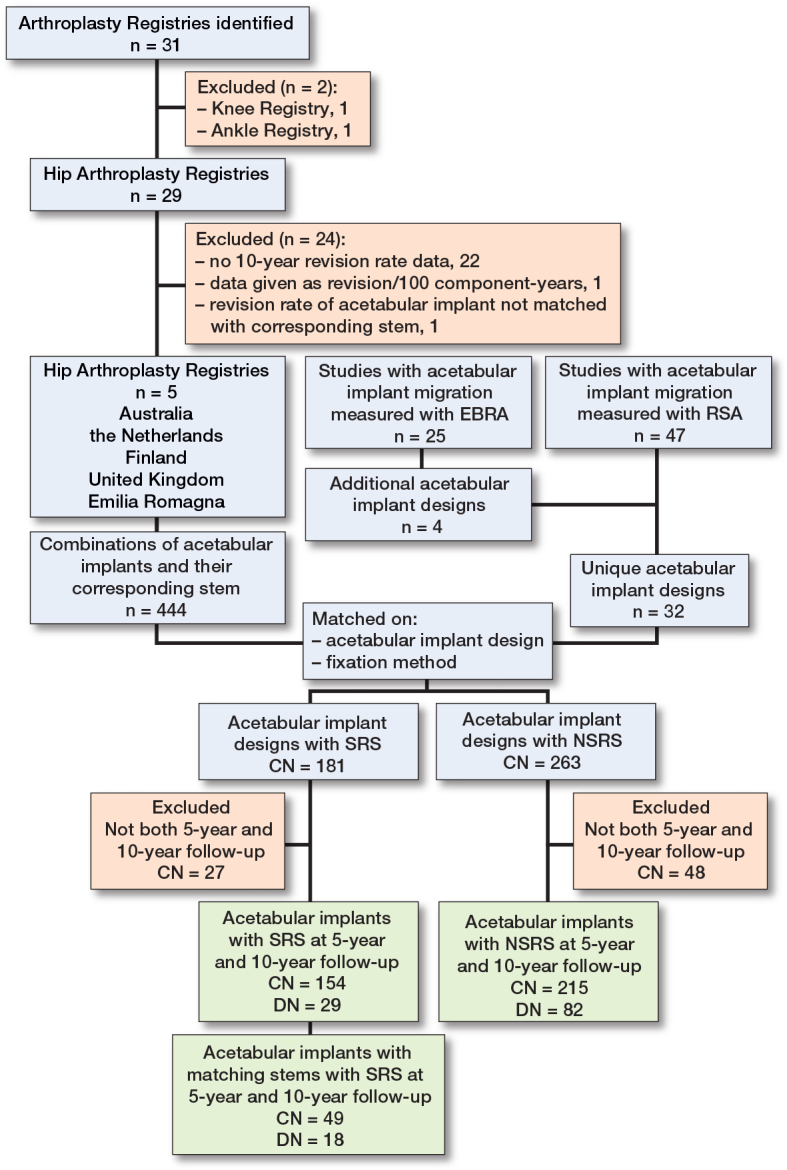
Number of registries and number of acetabular implant designs and combinations included in the study. CN = number of acetabular implant and stem combinations; DN = number of acetabular implant designs; SRS = sensitive radiographic surveillance; NSRS = no sensitive radiographic surveillance.

**Table 1:** The 3 most used THA acetabular implant and femoral stem combinations with 10-year revision data (acetabular implant, femoral stem) from the 5 included registry annual reports

Implant groups	Australia [18] 2022 n = 796,686	Netherlands [19] 2022 n = 503,563	Finland [20] 2022 n = 250,045	United Kingdom [21] 2022 n = 1,614,242	Emilia Romagna [22] 2020 n = 137,612
1	Trident (Shell),	Pinnacle,	Exeter Universal,	Pinnacle,	Fixa TI-por,
	Exeter V40	Corail	Exeter	Corail	Apta
			Contemporary		
2	Pinnacle,	Allofit,	Summit,	Trident,	Fixa TI-por,
	Corail	Taperloc	Pinnacle Gription	Exeter V40	Hydra
		Complete			
3	Versafitcup CC,	Allofit,	Summit,	Exeter Contem-	AnCA Fit,
	Quadra-H	Alloclassic	Pinnacle	porary Flanged,	AnCA Fit
		Zweymuller SL		Exeter V40	

**Table 2:** Mean pooled revision rate (95% confidence interval) of acetabular implants with SRS vs NSRS at 5-year follow-up

Acetabular implants with	SRS			NSRS			Pooled mean
Registries	CN	DN	Revision rate (CI)	CN	DN	Revision rate (CI)	difference (CI)
Australia	53	16	3.1 (2.9–3.3)	51	27	4.2 (3.6–4.8)	0.8 (0.3 to 1.3)
Netherlands	28	14	3.1 (2.8–3.4)	38	26	2.9 (2.6–3.3)	–0.2 (–0.7 to 0.3)
Finland	32	14	4.2 (3.9–4.6)	61	37	5.2 (4.7–5.6)	0.8 (0.0 to 1.5)
United Kingdom	30	17	1.9 (1.6–2.2)	36	19	2.8 (2.5–3.1)	0.9 (0.4 to 1.3)
Emilia Romagna	11	8	2.4 (1.8–3.1)	29	18	3.1 (2.7–3.6)	0.7 (–0.1 to 1.5)
Combined registries	154	29	3.0 (2.8–3.1)	215	82	3.9 (3.7–4.2)	0.8 (0.5 to 1.1)

CN = number of acetabular implant and stem combinations;

DN = number of acetabular implant designs.

**Table 3:** Mean pooled revision rate (95% confidence interval) of acetabular implant and matching stems with SRS vs NSRS at 5-year follow-up

Acetabular implants with	SRS			NSRS			Pooled mean
Registries	CN	DN	Revision rate (CI)	CN	DN	Revision rate (CI)	difference (CI)
Australia	15	9	3.2 (2.9–3.5)	51	27	4.2 (3.6–4.8)	0.9 (–0.2 to 1.9)
Netherlands	7	6	3.6 (2.5–4.6)	38	26	2.9 (2.6–3.3)	-0.5 (–1.5 to 0.4)
Finland	10	9	3.9 (3.2–4.6)	61	37	5.2 (4.7–5.6)	1.2 (0.0 to 2.4)
United Kingdom	13	11	2.0 (1.7–2.4)	36	19	2.8 (2.5–3.1)	0.6 (0.1 to 1.2)
Emilia Romagna	4	4	2.1 (0.8–3.4)	29	18	3.1 (2.7–3.6)	1.1 (–0.1 to 2.2)
Combined registries	49	18	2.9 (2.6–3.2)	215	82	3.9 (3.7–4.2)	0.8 (0.3 to 1.3)

For abbreviations. see Table 2.

### Revision rates of acetabular implants with SRS vs NSRS at 5-year follow-up

Mean all-cause revision rates at 5 years for acetabular implants with SRS and NSRS were 3.0% (CI 2.8–3.1) and 3.9% (CI 3.7–4.2) respectively, with a pooled mean difference of 0.8% (CI 0.5–1.1) favoring acetabular implants with SRS. There was high heterogeneity in both SRS and NSRS cohorts across all registry reports (I2 = 96% for acetabular implants with SRS and I2 = 98% acetabular implants with NSRS) (Table 2).

### Revision rates of acetabular implant and matching stems with SRS vs NSRS at 5-year follow-up

Mean all-cause revision rates of acetabular implants with SRS and matching stems and NSRS at 5-years were 2.9% (CI 2.6–3.2) and 3.9% (CI 3.7–4.2) respectively, with a pooled mean difference of 0.8% (CI 0.3–1.3) in favor of acetabular implants with SRS (Table 3).

### Revision rates of acetabular implants with SRS vs NSRS at 10-year follow-up

Mean all-cause revision rates at 10 years for acetabular implants with SRS and NSRS were 5.2% (CI 4.9–5.5) and 7.4% (CI 7.0–7.9) respectively, with a pooled mean difference of 1.8% (CI 1.2–2.3) in favor of acetabular implants with SRS. There was high heterogeneity in both SRS and NSRS cohorts across all registry reports (I2 = 97% for acetabular implants with SRS and I2 = 98% NSRS) (Table 4).

**Table 4:** Mean pooled revision rate (95% confidence interval) of acetabular implants with SRS vs NSRS at 10-year follow-up

Acetabular implants with	SRS			NSRS			Pooled mean
Registries	CN	DN	Revision rate (CI)	CN	DN	Revision rate (CI)	difference (CI)
Australia	53	16	5.0 (4.7–5.4)	51	27	5.0 (4.4–5.5)	–0.3 (–0.9 to 0.3)
Netherlands	28	14	4.5 (4.0–5.0)	38	26	4.5 (4.0–5.0)	–0.1 (–0.9 to 0.6)
Finland	32	14	8.4 (7.4–9.4)	61	37	13 (11–14)	4.1 (1.9 to 6.2)
United Kingdom	30	17	3.2 (2.6–3.7)	36	19	5.5 (4.7–6.3)	2.2 (1.3 to 3.1)
Emilia Romagna	11	8	5.7 (4.3–7.0)	29	18	5.2 (4.5–5.9)	–0.4 (–1.7 to 1.0)
Combined registries	154	29	5.2 (4.9–5.5)	215	82	7.4 (7.0–7.9)	1.8 (1.2 to 2.3)

For abbreviations. see Table 2.

### Revision rates of acetabular implants and matching stems with SRS vs NSRS at 10-year follow-up

Mean all-cause revision rates of acetabular implants with SRS and matching stems and NSRS at 10 years were 5.3% (CI 4.8–5.9) and 7.4% (CI 7.0–7.9) respectively, with a pooled mean difference of 1.8% (CI 0.8–2.7) in favor of acetabular implants with SRS (Table 5).

**Table 5:** Mean pooled revision rate (95% confidence interval) of acetabular implant and matching stems with SRS vs NSRS at 10-year follow-up

Acetabular implants with	SRS			NSRS			Pooled mean
Registries	CN	DN	Revision rate (CI)	CN	DN	Revision rate (CI)	difference (CI)
Australia	15	9	5.6 (4.9–6.2)	51	27	5.0 (4.4–5.5)	–0.8 (–1.6 to 0.1)
Netherlands	7	6	5.7 (4.1–7.3)	38	26	4.5 (4.0–5.0)	–1.1 (–2.5 to 0.3)
Finland	10	9	8.2 (6.6–9.8)	61	37	13 (11–14)	4.3 (0.6 to 7.9)
United Kingdom	13	11	3.5 (2.7–4.3)	36	19	5.5 (4.7–6.3)	1.9 (0.6 to 3.3)
Emilia Romagna	4	4	4.2 (2.3–6.2)	29	18	5.2 (4.5–5.9)	1.0 (-0.9 to 2.9)
Combined registries	49	18	5.3 (4.8–5.9)	215	82	7.4 (7.0–7.9)	1.7 (0.8 to 2.7)

For abbreviations. see Table 2.

## Discussion

The aim of this study was to investigate whether acetabular implants with NSRS are associated with higher revision rates than those with SRS. We found that acetabular implants with NSRS are associated with higher pooled revision rates compared with acetabular implants with SRS across 5 arthroplasty registries. Although the mean difference of 1.8% in all cause revision rate at 10 years may seem relatively small, the results of our study should be interpreted relative to primary THA revision rates [10]. Considering the current international benchmark all-cause revision rate is approximately 5% at 10 years [10], an absolute increase of 1.8% would translate to a relative increase of 36% in the revision burden. Implants with NSRS were more likely to have revision rates greater than 5% at 10 years. Whilst our finding that implants monitored with NSRS are associated with higher revision rates, this does not imply causation. Despite investigating only 2 SRS methods, our findings support the literature that implants that have any published evidence may be associated with lower long-term revision rates [23]. The pooled differences between acetabular implants tested with SRS and NSRS in the Australian registry were less distinct at the 10-year timepoint relative to the 5-year, which may be influenced by the reduced number of patients included at longer-term follow-up.

The findings of the study suggest that acetabular implants that publicly reported SRS data performed better when examining their survivorship at a registry level. Differences between SRS and NSRS may be influenced by reporting bias. Underperforming implants may have undergone SRS testing, but their results were not published, leading to potential underreporting of unfavorable outcomes. As it is unclear how many implants were tested with SRS but not reported, this study highlights the importance of conducting and publishing SRS results for all implant designs. A possible explanation for the observed association is that SRS can detect early excessive migration of acetabular implants that are at risk of aseptic loosening. Early identification of poorly performing implants may lead to their withdrawal from the market, while well-studied, high-performing designs continue to be used, leading to lower revision rates. Alternatively, larger orthopedic medical device companies may have the financial resources to conduct expensive prospective RSA studies. This may bias SRS towards implants used in larger volumes that have undergone more preclinical testing and post-market surveillance. However, conducting SRS alone is not a causative factor for achieving lower revision rates.

The results of our study are comparable to a previous study by Hasan et al. [10], who found non-RSA-tested knee implants had a 1% higher revision rate at 10 years, equating to a relative increase of 20% of TKA revision burden. A key difference from the previous knee study is that our investigation of acetabular implants had to consider outcomes by acetabular implant and femoral stem combination. Within each THA, the acetabular implants and femoral stem used can be from different manufacturers, whereas in TKA, tibial and femoral implants are always matched. Our study performed further analysis of pooled revision rates for acetabular implants with matching stems as reported in EBRA and RSA studies. Importantly, our analysis showed similar reduced pooled all-cause revision rates for acetabular implants with SRS and matching stems at both 5-year and 10-year follow-up. Therefore, the inclusion of different stem combinations in our analysis did not substantially influence the comparative revision rates between the SRS and NSRS groups. Our findings are supported by a Dutch study including clinical evidence of implant performance, such as the use of an ODEP (Orthopaedic Data Evaluation Panel). This regulation may reduce the overall revision rate of all implants as more implants are likely to have clinical evidence of long-term success. Hoogervorst et al. found hip implants with an ODEP rating had lower revision rates at 3, 5, and 10-years’ follow-up [23]. Implants were assessed by ODEP based on the revision data from observational studies, which included single-center RSA and EBRA studies of implant stability. While our study focused specifically on long-term registry data and published radiographic measurements of implant stability, the findings by Hoogervorst et al. support the results and may have included additional surveillance methods [23].

### Strengths

A strength of this study is the inclusion of published EBRA and RSA studies as evidence of SRS. Unlike prospective RSA studies that require access to specialized equipment and expensive software to measure implant migration, EBRA studies require only access to plain anterior posterior pelvis radiographs taken routinely at clinical follow-up, which can be measured retrospectively. Thus, our findings may be more applicable to the broader orthopedic community as the recommendation for SRS is not limited to one specialized radiographic surveillance method.

Pooling a high volume of cases from 5 registries (Australia, Netherlands, Finland, United Kingdom, and Emilia Romagna), allowed observation of implant survivorship on a global scale. This minimized the impact of regional bias as there are implant usage preferences evident in each country (see Table 1). The combined pooled analysis of all registries revealed differences in the overall revision rates of acetabular implants tested with SRS and NSRS, which was not identified when examining smaller numbers of THAs in individual registries.

### Limitations

First, we were unable to investigate the revision rate of the acetabular implants independent of the femoral stem used. Available data obtained from national registries reported the all-cause revision rate by acetabular implant and femoral stem combination. Second, there may have been a delay between publication of SRS findings and implant introduction, the timing of which is varied and difficult to determine across several countries. Additionally, all the acetabular implants with SRS identified in the review may not have been studied with the intention of radiographic surveillance of new implant designs. This is indeed the case, as some of the implants identified in the RSA studies may have been used as the existing “gold standard” implant against which to compare new acetabular designs. For example, Salemyr et al. [24] compared a new acetabular implant with a porous titanium backside (Regenerex shell) with a clinically well-proven titanium acetabular implant (Pinnacle shell). Third, our study was unable to compare the revision rate due to aseptic loosening or other mechanisms of failure such as dislocation and infection, as revision data by diagnosis was not always available. As implant migration is a surrogate marker for predicting long-term revision due to aseptic loosening, it would have been better to assess the long-term loosening rate as a cause of revision. An improvement in all-cause revision rate between acetabular implants with SRS and NSRS is indicative of the clinical effectiveness of acetabular implants with evidence of SRS at both mid- and long-term follow-up. Therefore, it would be likely that an analysis restricted to revision due to loosening would demonstrate similar findings. Additionally, both the SRS and NSRS groups were identified and analyzed from the same publicly available national registry reports, covering similar implant types and time of introduction. Therefore, confounding factors are expected to affect both groups similarly. Fourth, other variables such as bearing surface, liner design, head size, date of implant introduction, or presence of screws were not included in registry data, or within this study. However, our previous systematic review showed that implant and surgical factors, including type of polyethylene and time of introduction, did not influence the migration patterns of acetabular implants [11]. Finally, the implants investigated in our study have been introduced over a long period of time and there were variabilities in the number and revision rates of the same implant designs across the different registries. As a result, there was evidence of heterogeneity with I2 values above 96%. The high heterogeneity of this study indicates that variation between hip arthroplasty registries cannot be explained by chance alone. Variation of revision rates may have been influenced by national guidelines and policies in implant introduction.

Digital radiographic measurement techniques continue to improve. This will encourage more studies of new implants with SRS. As an example, RSA methodology has improved [25] and more recently computed tomography RSA (CT-RSA) methods have been validated [26] where intraoperative insertion of tantalum markers and specialized radiographic examinations above a calibration cage are no longer required. Combined with the findings of our study, we recommend that all new acetabular implants should be tested with SRS prior to being introduced for widespread use in the orthopedic market.

### Conclusion

Acetabular implants with NSRS were associated with higher all-cause revision rates at both 5- and 10-years’ follow-up compared with acetabular implants with SRS. A relative increase in revision rate of approximately 1.8% at 10 years represents an increase of approximately 36% in revision burden, which impacts hospital services and health-related costs. We encourage that all implant designs be tested with SRS and the published results disseminated within the orthopedic community for critical evaluation prior to widespread use.

## Supplementary Material



## Supplementary data

Appendices 1–3 are available as Supplementary data on the article home page, doi: 10.2340/17453674.2026.45292

## Notes

SC held a Research Fellowship from the Hospital Research Foundation during the period of this study.

Conceptualization: CHC, SC, BP, JA. Methodology: CHC, JM, BP, SC. Validation: CHC. Formal analysis: CHC. Data curation: CHC, BP. Writing original draft: CHC. Writing review and editing: CHC, JA, DS, LS, CW, SC. Visualization: CHC, JA, LB, SC. Supervision: JA, BP, SC. Project administration: CHC, JA, BP, SC.

Handling co-editors: Keijo Mäkelä and Robin Christensen

*Acta* thanks Geir Hallan and Anne Lübbeke for help with peer review of this manuscript.
