# Supplementary material for: Association of acetabular implants with sensitive radiographic surveillance on revision rates: a study based on 5 hip arthroplasty registries
Source: Acta Orthop. 2026 Feb 3;97:60–6. doi: 10.2340/17453674.2026.45292 (PMC12869144; doi:10.2340/17453674.2026.45292)
Supplement: Supplementary file 1 [file ActaO-97-45292-s1.pdf]

Appendix 1: Search strategy to identify THR acetabular implants studied with EBRA

PubMed

(Arthroplasty, Replacement, Hip[mh] OR Hip Arthroplasty[tiab] OR Hip Replacement[tiab] OR THR[tiab] Acetabular cup[tiab] Acetabular component[tiab] OR cup[tiab] OR cups[tiab] OR acetabular[tiab] OR liner[tiab] OR polyethylene[tiab]) AND (Migration[tiab] OR Migrat*[tiab] OR movement [tiab] OR rotat*[tiab] OR inclination[tiab] OR translat*[tiab] OR micromotion [tiab]) AND (Ein Bild Röntgen Analyse [mh] OR EBRA[tiab])

Embase

(hip arthroplasty or hip replacement or hip arthroplasty or hip replacement) AND (THR acetabular component or cup or cups or acetabular or liner or polyethylene) AND (Migration OR Migrat OR movement OR rotation OR inclination OR translation OR micromotion) AND (Ein Bild Rontgen Analyse OR EBRA)

Scopus

(hip arthroplasty OR hip replacement OR total hip replacement OR thr) AND (acetabular component OR cup OR cups OR acetabular OR liner OR poly-ethylene) AND (migrat* OR movement OR rotat* OR inclination OR translat* OR micromotion) AND (EBRA or Einzel Bild Rontgen Analyse or Ein Bild Rontgen Analyse)

Appendix 2: PRISMA flowchart of the systematic review of primary THR acetabular implants studied with EBRA

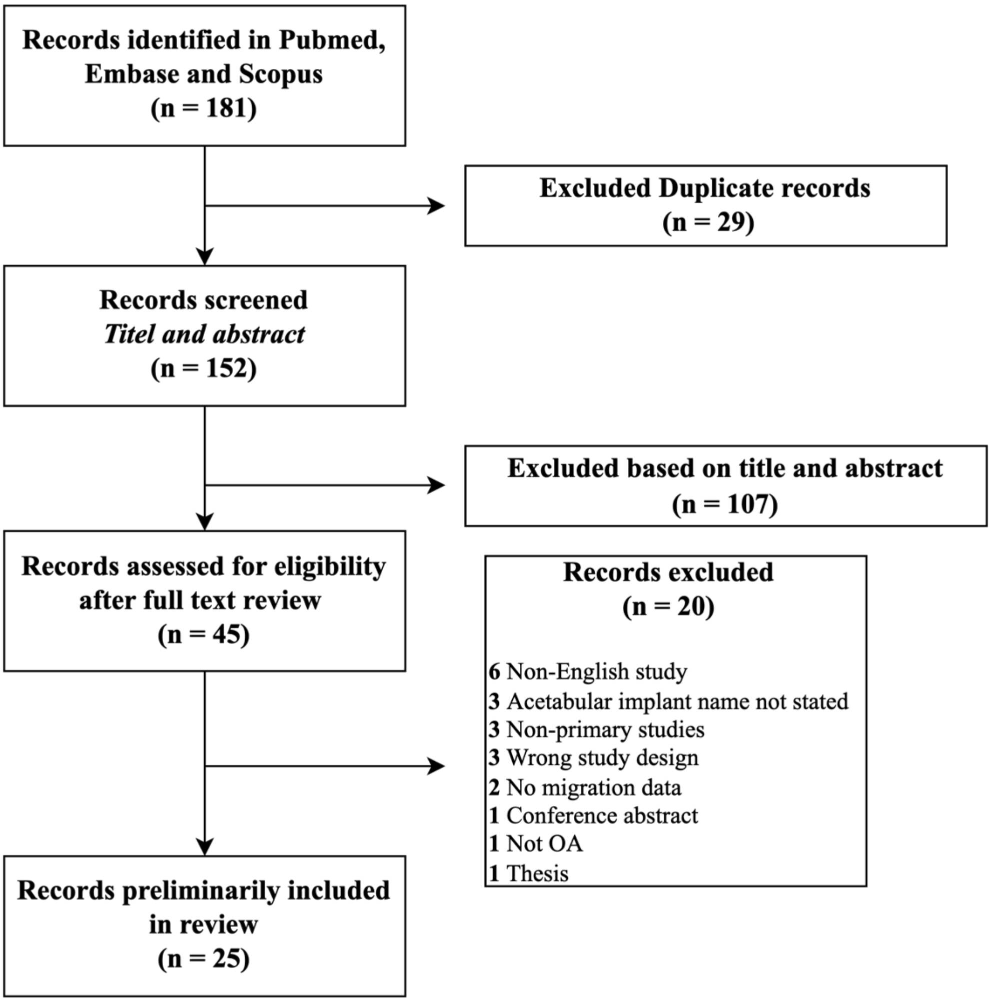

Appendix 3: The inclusion and exclusion criteria for the systematic review of THR acetabular implants studied with EBRA

The inclusion criteria were any studies that reported: (1) primary THR, (2) migration measured using EBRA and (3) the migration of the acetabular implant in any direction. Studies were excluded if they did not report acetabular implant migration; reported on fewer than 5 acetabular cups; acetabular implant migration was not measured using EBRA; the cohorts integrated the migration of acetabular cups used at revision THR; the studies were conference proceedings, thesis manuscript, or used languages not spoken by the review team; the studies

used in vitro or animal models; the studies did not separate acetabular cup migration via implant design
